# Factors Influencing Tooth Sensitivity: Insights From the Hospital Universiti Sains Malaysia Using Bootstrap-Enhanced Ordinal Regression

**DOI:** 10.7759/cureus.64641

**Published:** 2024-07-16

**Authors:** Muhamamd Amirul Mat Lazin, Wan Nazlee Wan Zainon, Arsalan Humayun, Ashwini M Madawana, Akram Hassan, Yu Zhang, Mohamad Arif Awang Nawi

**Affiliations:** 1 Department of Oral Medicine, School of Dental Sciences, Universiti Sains Malaysia (USM), Kota Bharu, MYS; 2 Department of Family Medicine, School of Dental Sciences, Universiti Sains Malaysia (USM), Kota Bharu, MYS; 3 Department of Dentistry, School of Dental Sciences, Universiti Sains Malaysia (USM), Kota Bharu, MYS; 4 Department of Epidemiology and Public Health, School of Dental Sciences, Universiti Sains Malaysia (USM), Kota Bharu, MYS

**Keywords:** dentin hypersensitivity, tooth sensitivity, dental epidemiology, bootstrap techniques, ordinal regression

## Abstract

Introduction

Tooth sensitivity, or dentin hypersensitivity (DH), is characterized by sharp, sudden pain in response to stimuli such as cold, heat, sweet, or acidic foods and drinks. In Malaysia, there is limited understanding of the epidemiological aspects of tooth sensitivity, necessitating focused research. The condition results from the exposure of dentinal tubules transmitting stimuli to nerves within the pulp, with contributing factors including gingival recession, enamel erosion, and periodontal disease. This study aims to investigate the factors associated with tooth sensitivity among patients at the Hospital Universiti Sains Malaysia (USM) using advanced statistical methods.

Methods

This study employed a computational research design to develop an ordinal regression and bootstrap methodology using the RStudio software (Posit PBC, Boston, MA) to analyze secondary data from the Hospital Universiti Sains Malaysia. Six variables were analyzed: tooth wear severity, patient's age, gender, smoking status, alcohol status, and type of toothbrush. The study was conducted in three phases: 1) the development of an ordinal regression model, 2) the development of algorithms for ordinal regression and bootstrap method, and 3) validation using tooth sensitivity data.

Results

The analysis revealed that the replication with 1000 samples provided the most precise estimates with small standard errors (SE) and consistently significant effects across variables. Tooth sensitivity was influenced by age, toothpaste type, toothbrush type, and brushing frequency.

Conclusion

The study highlights the importance of considering multiple variables such as age, toothpaste type, toothbrush type, and brushing frequency in understanding tooth sensitivity. The combined ordinal regression and bootstrap technique significantly improved the model's accuracy, providing valuable insights for dental health professionals. These findings underscore the need for specific guidelines on oral hygiene practices to manage and reduce the risk of tooth sensitivity.

## Introduction

Tooth sensitivity, or dentin hypersensitivity (DH), is a common dental problem characterized by sharp and sudden pain in response to stimuli that should not cause pain, such as cold, heat, sweet, or acidic foods and drinks [1]. Globally, the prevalence of tooth sensitivity varies widely, reported to affect between 4% and 74% of the population, depending on the diagnostic criteria and the population studied [2]. In Malaysia, the understanding of tooth sensitivity, particularly its epidemiological aspects, remains understudied, necessitating focused research to better understand and manage this condition.

The pathophysiology of tooth sensitivity involves the exposure of dentinal tubules that transmit stimuli to nerves within the pulp, resulting in pain [3]. Several factors contribute to this condition, including gingival recession, enamel erosion, and periodontal disease, which can expose dentinal tubules over time [4]. Despite its prevalence and impact on the quality of life, tooth sensitivity is often underreported and can be a significant challenge in dental practice, affecting patients' eating habits, oral hygiene practices, and overall quality of life [5].

Research into the prevalence and determinants of tooth sensitivity in specific populations, such as those attending the Hospital Universiti Sains Malaysia (USM), is crucial. It allows for the identification of high-risk groups and the development of effective prevention and management strategies [6]. This study aims to use advanced statistical methods, specifically ordinal regression with bootstrap techniques, to analyze the factors associated with tooth sensitivity in this population. Such approaches are particularly useful in handling the ordered categorical nature of data typically obtained in studies on tooth sensitivity severity [7].

## Materials and methods

This study utilized a computational research design focusing on the development of an ordinal regression and bootstrap methodology using the RStudio software (Posit PBC, Boston, MA) to analyze secondary data from the Hospital Universiti Sains Malaysia. The data included information on tooth sensitivity, which was used to build and assess the accuracy of statistical models. This study is composed of six variables such as tooth wear severity, patient's age, patient's gender, smoking status, alcohol status, and type of toothbrush (Table 1).

**Table 1:** Description of data among patients with tooth sensitivity

Number	Variables	Explanation of user variables
1	Tooth sensitivity	1 = Mild
		2 = Moderate
		3 = Severe
2	Age	1 = 20-39
		2 = 40-59
		3 = Above 59
3	Types of toothpaste	1 = Fluoridated
		2 = Non-fluoridated
		3 = Abrasive
		4 = Do not know
4	Types of toothbrush	1 = Soft
		2 = Medium
		3 = Hard
5	Frequency of brushing	1 = Once a day
		2 = Twice a day
		3 = More than twice
		4 = After meal
6	Method of brushing	1 = Horizontal
		2 = Vertical
		3 = Circular
		4 = Combination

**Figure 1:**
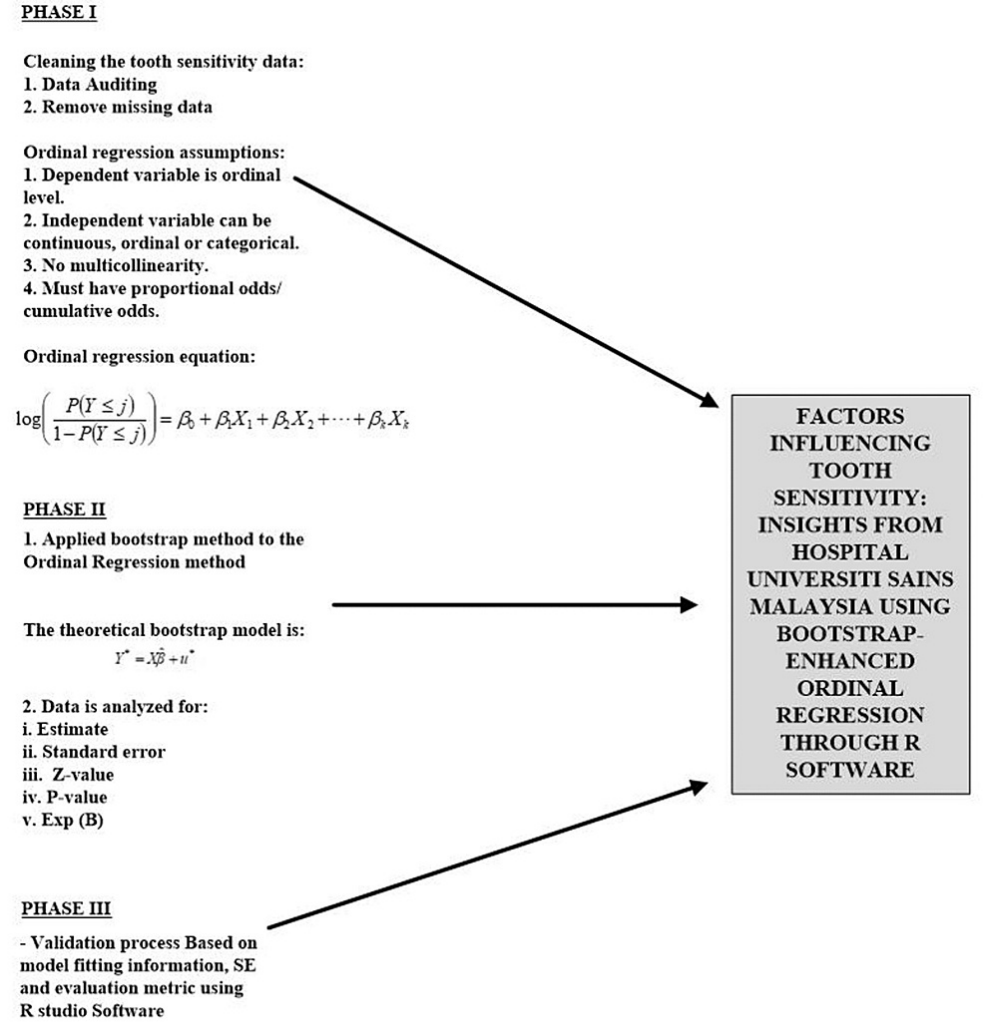
Methodology development of alternative model

Methodology development focuses on the application of health science modelling techniques to small sample sizes. In order to construct this model, it is necessary to translate the fundamental theory into a particular computer language. The study consists of four distinct phases, which are illustrated in Figure 1.

Phase I: The development of an algorithm for the ordinal regression model using the R software

The initial phase of our study technique involves the creation of an algorithm for the ordinal regression model using the R software (R Foundation for Statistical Computing, Vienna, Austria), which serves as the basis for our research. This phase is devoted to thoroughly characterizing the dataset, with each variable being evaluated on an ordinal scale. To do ordinal regression analysis in RStudio, a powerful platform for statistical computing and graphics, it is necessary to carefully configure the data. This involves not only ensuring that the data file is accurately located in the working directory but also precisely specifying the order of levels in the dependent variable along with the corresponding independent variables.

Phase I is characterized by the smooth integration of data preparation and analytical execution, supported by the general formula of ordinal logistic regression. This formula ensures that the levels of the dependent variable are accurately ordered and the independent variables are precisely defined, establishing a strong foundation for the research's advancement. The general formula for the ordinal logistic regression can be denoted as $log\left ( \frac{P(Y\leq j)}{1-P(Y\leq j)} \right )= \beta 0+\beta 1X_1+\beta 2 X_2+\cdot \cdot \cdot +\beta k X_k$ where *P(Y≤j)* is the cumulative probability of the response variable *Y* being in category* j *or lower, *β 0* is the intercept term specific to category *j,* and *β 1 β 2...β k* are the coefficients for the predictor variables *X*_1_,* X*_2_...*X_k_*.

Phase II: The development of algorithms for ordinal regression + bootstrap method using the R software

In phase II, the combination of ordinal regression and bootstrap technique was performed using the RStudio software to improve the accuracy of essential solutions. This study could also detect the outliers by using odds ratio (OR) coefficients for k category responses. Barnett and Lewis [8] discuss the bootstrap related to checking the modelling assumption in the face of outliers. This technique is improved by scaling the regression coefficients to their means [9].

Agresti [10] states that ordinal regression methods that are often used for ordinal scale response variables are to form logit functions from cumulative odds. She discusses the bootstrap as it can be applied to categorical data. The bootstrap methods began with original data or samples taken from the population and then calculated sample statistics. The next step is to copy the original sample several times to create a pseudo population with replacement using the empirical density function (EDF) [11].

The bootstrap method entails iteratively resampling the initial dataset with replacement to generate several pseudo samples. Subsequently, each of these samples is utilized to calibrate an ordinal regression model, and the distribution of the resulting coefficients across all bootstrap samples is examined. This technique has two purposes: First, it helps estimate the correctness of the regression coefficients, and second, it improves the overall reliability of the model by taking into account uncertainties in the sample data.

The general bootstrap algorithm for improving an ordinal regression model can be summarized as follows: 1) initialization, 2) bootstrap sampling, 3) model fitting, and 4) analysis.

Initialization

Let the original dataset have *N* observations. Decide on the number of bootstrap samples, *B*, typically a large number like 1000 or 2000, to ensure statistical reliability.

Bootstrap Sampling

For each bootstrap iteration, b=1, 2,...,B. Randomly sample N observations with replacement from the original dataset to create a bootstrap sample.

Model Fitting

For each bootstrap sample, fit the ordinal regression model to the sample, and extract and store the estimated coefficients and any other statistics of interest (e.g., the standard error {SE} of estimates).

Analysis

For each coefficient and statistic, calculate the standard error and other relevant summary statistics across all B bootstrap samples. These summary statistics provide an empirical distribution that reflects the sampling variability and allows for more robust inference. The formula for an ordinal regression model, specifically the proportional odds model, which is commonly used, can be represented as $log\left ( \frac{P(Y\leq j)}{1-P(Y\leq j)} \right )= \beta 0+\beta 1X_1+\beta 2 X_2+\cdot \cdot \cdot +\beta k X_k$ where *P(Y≤j)* is the cumulative probability of the response variable *Y* being in category *j* or lower, *β0* is the intercept term specific to category *j*, and *β1, β2...βk* are the coefficients for the predictor variables *X_1_, X_2_...X_k_*.

The bootstrap enhancement allows for a more nuanced understanding of how the coefficients behave across different samples drawn from the original dataset, providing insights into their variability and reliability.

Phase III: Validation process using tooth sensitivity data determined using the R software

In phase III, the focus shifts toward validating the combined ordinal regression and bootstrap model (alternative model) developed in phase II. This validation is critical to ensure the model's efficacy and reliability when applied to new or unseen data. A comprehensive evaluation involves several model fitting indices and pseudo R-squared measures, each providing unique insights into the model's performance. The comparison between the alternative and ordinal regression methods will be based on model fitting information (log-likelihood-final model), standard error, and evaluation metric (Akaike information criterion {AIC}, Bayesian information criterion {BIC}, McFadden's pseudo R-squared, Nagelkerke's {Cragg and Uhler} R-squared, Cox and Snell {ML}, and Nagelkerke {Cragg and Uhler}).

Ethical considerations

Ethical approval was obtained from the Human Research Ethics Committee of the USM Board (approval number: USM/JEPeM/19090548).

## Results

Phase I: Data analysis using ordinal regression method

The data from the tooth sensitivity case study at the Hospital Universiti Sains Malaysia was analyzed using an ordinal regression method. The analysis entailed a comparison between two models: an "intercept only" model and a "final" model that incorporates predictors. The chi-square test revealed a statistically significant disparity in model fit, as indicated by a chi-square value of 35.803 and a p-value of less than 0.0001 (Table 2). This suggests that the model using predictors significantly enhances the accuracy compared to the model that merely includes the intercept. The results strongly indicate that the predictors used in the final model are highly helpful in elucidating or forecasting dental sensitivity among the patients.

**Table 2:** Model fitting information ***Significant level at <0.0001 df: degrees of freedom

Model	Log-likelihood	Chi-square	df	Significance
Intercept only	32.667	35.803	12	0.000***
Final	14.765			

Table 3 shows the associations between various factors and tooth sensitivity conditions categorized as mild, moderate, and severe using ordinal regression analysis. The analysis indicates that neither the 40-59 age group (B = -2.178, SE = 2.443, p-value = 0.373, and Exp (B) = 0.113) nor the 60 and above age group (B = -4.333, SE = 2.837, p-value = 0.127, and Exp (B) = 0.013) displays statistically significant effects on tooth sensitivity. The low odds ratios of 0.113 and 0.013 suggest that age within these brackets does not substantially impact the likelihood of experiencing tooth sensitivity. The type of toothpaste used does not seem to significantly affect tooth sensitivity. None of the toothpaste categories exhibited statistically significant impacts, as indicated by their p-values. This suggests that the choice of toothpaste among these options does not notably influence tooth sensitivity levels.

**Table 3:** Ordinal regression model *Significant level at <0.05

Variable	Estimate	Standard error	z-value	Significance	Exp (B)
Age					
40-59	-2.178	2.443	-0.892	0.373	0.113
60 and above	-4.333	2.837	-1.527	0.127	0.013
Toothpaste					
Fluoridated	-1.982	2.195	-0.903	0.366	0.138
Abrasive	3.894	2.285	1.704	0.088	49.083
Do not know	-4.264	2.321	-1.837	0.066	0.014
Toothbrush					
Medium	4.904	1.989	2.465	0.013*	134.844
Hard	3.492	2.012	1.736	0.083	32.857
Frequency of brushing					
Twice a day	-1.190	1.712	-0.695	0.487	0.304
More than twice	3.793	1.977	1.918	0.055	44.407
Method of brushing					
Vertical	-8.682	4.857	-1.788	0.074	0.000
Circular	-3.049	2.529	-1.205	0.228	0.047
Combination	-5.249	2.587	-2.029	0.043*	0.005
Tooth sensitivity					
Mild|moderate	-6.775	3.384	-2.002	0.045*	0.001
Moderate|severe	-1.433	2.291	-0.626	0.531	0.238

Using a medium toothbrush shows a statistically significant positive effect on tooth sensitivity. Individuals using a medium toothbrush have significantly higher odds (approximately 135 times) of experiencing increased tooth sensitivity compared to the soft toothbrush (B = 4.904, SE = 1.989, p-value = 0.013, and Exp (B) = 134.844). Meanwhile, using a hard toothbrush also seems to positively influence tooth sensitivity, albeit not reaching statistical significance. The odds ratio of 32.857 indicates a relatively heightened likelihood of experiencing higher tooth sensitivity compared to the soft toothbrush (B = 3.492, SE = 2.012, p-value = 0.083, and Exp (B) = 32.857).

Neither brushing frequency category, twice a day nor more than twice a day, exhibits a significant effect on tooth sensitivity. Their p-values, exceeding 0.05, suggest that the frequency of brushing within these categories does not significantly impact tooth sensitivity levels. Using a vertical brushing method trends toward a negative impact on tooth sensitivity, although this trend is not statistically significant, while the combination method shows a trend toward significance with a p-value of 0.043 (B = -5.249, SE = 2.587, p-value = 0.043, and Exp (B) = 0.005). The odds ratio (Exp (B)) of 0.005 implies that individuals utilizing this combined brushing method have substantially lower odds (approximately 0.5%) of experiencing higher tooth sensitivity compared to those employing the standard or reference brushing method. It suggests a notable reduction in the likelihood of higher tooth sensitivity among individuals using this combined technique.

Phase II: The development of algorithms for ordinal regression + bootstrap method (alternative method)

Table 4 shows the comparison across replications (100, 300, 500, and 1000) in the ordinal regression with the bootstrap method; each replication offers varying degrees of precision and significance in estimating the impact of different factors on tooth sensitivity.

**Table 4:** Alternative model (ordinal regression + bootstrap method) *Significant level at <0.05 **Significant level at <0.001 ***Significant level at <0.0001

Replication	Variable	Estimate	Standard error	z-value	Significance	Exp (B)
100	Age					
	40-59	-2.873	1.335	-2.152	0.031*	0.057
	60 and above	-4.914	1.647	-2.985	0.003**	0.007
	Toothpaste					
	Fluoridated	-1.673	1.417	-1.181	0.238	0.188
	Abrasive	4.345	1.449	2.999	0.003**	77.073
	Do not know	-5.356	1.608	-3.330	0.000***	0.005
	Toothbrush					
	Medium	6.171	1.447	4.265	0.000***	478.79
	Hard	2.473	1.396	1.772	0.076	11.855
	Frequency of brushing					
	Twice a day	-2.797	1.217	-2.299	0.022*	0.061
	More than twice	3.255	1.346	2.418	0.016*	25.913
	Method of brushing					
	Vertical	-12.329	3.594	-3.431	0.000***	0.000
	Circular	-4.791	1.774	-2.701	0.007**	0.008
	Combination	-6.854	1.851	-3.703	0.000***	0.001
	Tooth sensitivity					
	Mild|moderate	-9.779	2.598	-3.763	0.000***	0.000
	Moderate|severe	-3.584	1.762	-2.035	0.042*	0.028
300	Age					
	40-59	-3.517	0.841	-4.183	0.000***	0.03
	60 and above	-5.543	1.005	-5.512	0.000***	0.004
	Toothpaste					
	Fluoridated	-1.487	0.825	-1.802	0.071*	0.226
	Abrasive	3.897	0.854	4.563	0.000***	49.269
	Do not know	-5.407	0.913	-5.920	0.000***	0.004
	Toothbrush					
	Medium	6.128	0.741	8.272	0.000***	458.328
	Hard	3.268	0.730	4.477	0.000***	26.263
	Frequency of brushing					
	Twice a day	-0.915	0.631	-1.450	0.147	0.40
	More than twice	4.220	0.704	5.994	0.000***	68.041
	Method of brushing					
	Vertical	-9.426	1.837	-5.131	0.000***	0.00008
	Circular	-2.807	0.931	-3.016	0.003**	0.06
	Combination	-6.235	0.951	-6.555	0.000***	0.002
	Tooth sensitivity					
	Mild|moderate	-7.757	1.271	-6.103	0.000***	0.0004
	Moderate|severe	-1.905	0.870	-2.189	0.029*	0.149
500	Age					
	40-59	-2.142	0.669	-3.199	0.001**	0.117
	60 and above	-4.5832	0.801	-5.674	0.000***	0.01
	Toothpaste					
	Fluoridated	-2.279	0.561	-4.061	0.000***	0.102
	Abrasive	3.982	0.617	6.457	0.000***	53.64
	Do not know	-4.798	0.661	-7.255	0.000***	0.008
	Toothbrush					
	Medium	5.528	0.570	9.693	0.000***	251.538
	Hard	4.047	0.578	7.004	0.000***	57.2
	Frequency of brushing					
	Twice a day	-1.065	0.458	-2.324	0.020*	0.345
	More than twice	4.637	0.542	8.556	0.000***	103.179
	Method of brushing					
	Vertical	-9.746	1.365	-7.139	0.000***	0.0001
	Circular	-3.820	0.716	-5.338	0.000***	0.022
	Combination	-6.468	0.735	-8.805	0.000***	0.002
	Tooth sensitivity					
	Mild|moderate	-7.723	0.965	-8.004	0.000***	0.0004
	Moderate|severe	-1.611	0.633	-2.546	0.011*	0.199
1000	Age					
	40-59	-2.04	0.468	-4.363	0.000***	0.130
	60 and above	-4.641	0.532	-8.727	0.000***	0.01
	Toothpaste					
	Fluoridated	-1.844	0.419	-4.402	0.000***	0.158
	Abrasive	4.799	0.445	10.778	0.000***	121.33
	Do not know	-4.525	0.438	-10.333	0.000***	0.011
	Toothbrush					
	Medium	5.250	0.379	13.853	0.000***	190.59
	Hard	3.561	0.376	9.471	0.000***	35.199
	Frequency of brushing					
	Twice a day	-1.413	0.313	-4.522	0.000***	0.243
	More than twice	4.187	0.370	11.310	0.000***	65.849
	Method of brushing					
	Vertical	-9.532	0.902	-10.563	0.000***	0.0001
	Circular	-3.0116	0.466	-6.463	0.000***	0.049

Replication 100

For this replication, the results show that both age groups ("40-59" and "60 and above") have negative estimated coefficients for tooth sensitivity, indicating a detrimental impact. Only the "60 and above" age group has a statistically significant impact. Toothpaste types such as "abrasive" show a significantly positive impact on tooth sensitivity, while "do not know" has a significantly negative effect. Medium toothbrushes display a significantly positive effect, and brushing more than twice a day also shows a significant positive impact. Methods of brushing such as "vertical" and "combination" have significant negative effects on tooth sensitivity.

Replication 300

Similar trends are observed in this replication compared to replication 100, with both age groups ("40-59" and "60 and above") displaying negative effects on tooth sensitivity, but with increased statistical significance. The impacts of toothpaste types, toothbrushes, brushing frequency, and brushing methods maintain consistency with improved statistical significance in some cases.

Replication 500

In this replication, similar trends persist in the negative effects of both age groups on tooth sensitivity but with slightly improved precision and significance. Toothpaste types, toothbrushes, brushing frequency, and brushing methods continue to display consistent impacts, with some improvements in precision and significance levels.

Replication 1000

Consistent negative effects on tooth sensitivity for both age groups are observed with increased precision and highly significant impacts. Similar trends in toothpaste, toothbrush types, brushing frequency, and methods of brushing are observed with higher precision in estimates, maintaining their significance. The effects appear to be more pronounced and precise in this replication compared to the others. In deciding the best replicate for this case study II, replication 1000 generally offers the most precise estimates and provides small standard error and consistently significant effects across most variables, suggesting a higher confidence level in the results compared to the other replications.

Phase III: Comparison between ordinal regression and alternative method based on model fitting information, standard error, and evaluation metric

The log-likelihood value for the typical ordinal regression model is 14.765. When the number of replications grows from 100 to 1000 in the alternative ordinal regression model, the log-likelihood values experience significant increases of 42.833, 128.78, 213.21, and 478.53, respectively (Table 5). The log-likelihood values for the alternative ordinal regression model with more replications exhibit a significant increase compared to the log-likelihood value for the standard ordinal regression model. The alternative models have higher log-likelihood values, indicating a superior fit or a greater likelihood of the observed data as compared to the standard ordinal regression model. This suggests that the alternative model, particularly with a greater number of replications, has a superior fit or more accurately reflects the observed data for tooth sensitivity among patients in the Hospital Universiti Sains Malaysia in comparison to the standard ordinal regression model.

**Table 5:** Evaluation metrics of the ordinal regression and alternative ordinal regression model for case study II

Indicator	Ordinal regression method	100	300	500	1000
Model fitting information (log-likelihood final model)	14.765	42.833	128.78	213.21	478.53
Standard error	9.864	6.918	3.668	2.807	1.844
Akaike information criterion (AIC)	57.531	113.667	285.553	454.422	959.712
Bayesian information criterion (BIC)	77.148	150.139	337.406	513.426	1028.42
McFadden's pseudo R-squared	0.548	0.605	0.607	0.608	0.572
Nagelkerke's R-squared	0.548	0.605	0.607	0.608	0.572
Cox and Snell (ML)	0.697	0.731	0.734	0.734	0.712
Nagelkerke (Cragg and Uhler)	0.786	0.826	0.827	0.828	0.804

The standard error of the conventional ordinal regression model is 9.864. Increasing the number of replications from 100 to 1000 in the alternative ordinal regression model results in a decrease in standard error values of 6.918, 3.668, 2.807, and 1.844, respectively (Table 5). The standard error values for the alternative ordinal regression model with increased replications exhibit a significant reduction compared to the standard error value for the standard ordinal regression model. Reduced standard errors indicate increased precision or decreased variability in the estimations derived from the model. This suggests that the alternative model, particularly with a greater number of replications, yields more accurate estimates or reduces the level of uncertainty in estimates for tooth sensitivity among patients at the Hospital Universiti Sains Malaysia, as compared to the standard ordinal regression model.

For the ordinal regression model, the AIC is 57.531, indicating the quality of fit of this model to the data regarding tooth sensitivity. As the number of replications in the alternative ordinal regression models increases (from 100 to 1000), the AIC values also rise gradually, from 113.667 to 959.712. Higher AIC values suggest a decreasing quality of fit between the model and the data. Therefore, based on the AIC values alone, the ordinal regression model seems to provide a better fit to the data regarding tooth sensitivity. For the ordinal regression model, the BIC is 77.148, signifying the balance between model fit and complexity for this specific model in capturing tooth sensitivity data. As the number of replications in the alternative ordinal regression models increases (from 100 to 1000), the BIC values also increase progressively, from 150.139 to 1028.42. Higher BIC values suggest a decreasing model quality or a poorer trade-off between fit and complexity. Therefore, based on the BIC values, the ordinal regression model seems to provide a better balance between goodness of fit and model complexity.

For the ordinal regression model, McFadden's pseudo R-squared and Nagelkerke's R-squared is 0.548, indicating the proportion of variance in tooth sensitivity explained by this specific model. As the number of replications in the alternative ordinal regression models increases (from 100 to 500), McFadden's pseudo R-squared and Nagelkerke's R-squared values show a slight increase, reaching 0.608 for 500 replications. This suggests a slight improvement in the models' explanatory power. Overall, the ordinal regression model initially shows a reasonable level of explanatory power. The alternative models display some improvement up to 500 replications, but at 1000 replications, there is a slight decrease in explanatory power compared to the model with 500 replications.

For the ordinal regression model, the Cox and Snell (ML) pseudo R-squared is 0.697, signifying the proportion of explained variance in tooth sensitivity by this specific model. As the number of replications in the alternative ordinal regression models increases (from 100 to 500), the Cox and Snell (ML) values exhibit a slight increase, plateauing at 0.734 for 300 and 500 replications. This suggests a modest improvement in the models' explanatory power. Overall, the ordinal regression model initially shows a decent level of explanatory power. The alternative models demonstrate some improvement up to 500 replications.

Nagelkerke's R-squared, using the Cragg and Uhler formula, evaluates the proportion of variance explained by a model relative to a perfect model. For the ordinal regression model, the Nagelkerke (Cragg and Uhler) R-squared is 0.786, indicating the amount of explained variance in tooth sensitivity by this specific model. As the number of replications in the alternative ordinal regression models increases (from 100 to 500), the Nagelkerke (Cragg and Uhler) R-squared values show a gradual increase, reaching 0.828 for 500 replications. This suggests an enhancement in the models' explanatory power. However, there is a slight decrease in the Nagelkerke (Cragg and Uhler) R-squared for the alternative model with 1000 replications, dropping to 0.804. Overall, the ordinal regression model initially demonstrates a good level of explanatory power.

## Discussion

The results of our study support the correlation between using a medium toothbrush and experiencing heightened tooth sensitivity, which is consistent with the findings of Blaizot et al. in 2020 [12]. Blaizot et al. found a significant association between the usage of a medium brush or an unspecified type of brush and a higher occurrence of tooth sensitivity, in comparison to other toothbrush types such as soft or electronic brushes [12]. The outcomes of our research confirm the noteworthy influence of employing combined brushing techniques on tooth sensitivity. This observation is corroborated by Ali et al.'s 2022 study [13], which established a robust correlation between utilizing both horizontal and vertical brushing methods and the occurrence of tooth sensitivity and cervical lesions. This supports the idea that incorrect brushing methods, such as the combination approach, can cause both tooth sensitivity and abrasion.

The current study investigated the complex correlation between tooth sensitivity and abrasion, which is consistent with the research conducted by Kumar et al. in 2016 [6] and Yadav et al. in 2012 [14]. A significant finding arose from this inquiry, suggesting that a considerable proportion of persons with cervical abrasion also reported the occurrence of dental hypersensitivity. Hypersensitive cervical abrasion is a difficult dental condition that causes substantial discomfort and has a dramatic impact on the daily lives of those affected. Furthermore, the study did a detailed analysis of individual teeth, uncovering that the connection between sensitivity and abrasion was mostly detected in the premolars of the upper jaw. The particular group of teeth showed a strong correlation between tooth sensitivity and abrasion. Significantly, the unequal spread of hypersensitive cervical abrasion across several tooth categories emphasizes the need for a customized and thorough assessment and treatment strategy that considers the distinct characteristics of each tooth type.

After implementing the bootstrap method (100, 300, 500, and 1000 replications) in the ordinal regression model, replication 1000 generally offers the most precise estimates and provides small standard error and consistently significant effects across most variables. Therefore, replication 1000 shows that all predictors such as age, types of toothpaste, types of toothbrush, frequency of brushing, and method of brushing significantly contribute to tooth sensitivity among patients in the Hospital Universiti Sains Malaysia. The findings of this study are in consensus with the results of other research studies conducted by Sud in 2015 [15], Hegde and Nireeksha [16], and Amin et al. in 2018 [17]. These studies have consistently reported a trend indicating that dentine sensitivity becomes more prevalent as individuals age. The rise in dentine sensitivity as individuals age can be ascribed to the gradual impact on tooth structure over time, leading to a heightened degree of structural tooth deterioration. Over time, the teeth can deteriorate due to factors such as abrasive substances and mechanical forces when chewing, which can damage the protective layers of enamel and dentin. The progressive erosion of tooth structure results in the increased exposure of the dentin, rendering it more vulnerable to external stimuli and hence heightening the likelihood of dentin sensitivity.

Furthermore, the study's age-specific analysis indicated that persons aged approximately 40 and older displayed a significantly elevated prevalence of dentine sensitivity. This discovery aligns with analogous inquiries carried out by Haneet and Vandana in 2016 [18]. The consistent findings across several research and populations highlight the strength and reliability of the observed link between age and dentine sensitivity, especially among older individuals. Moreover, the data obtained from this study on dentine sensitivity largely aligns with the results reported by Bartlett et al. in 2011 [19], where 58% of the participants in their study demonstrated dentine hypersensitivity. The consistency of these findings across multiple research provides more evidence for the widespread occurrence of dentine sensitivity as a significant issue in the field of dental healthcare.

Recent clinical investigations consistently confirm the efficacy of fluoride toothpaste in controlling dentin hypersensitivity. A comprehensive investigation conducted by Hu et al. [20] revealed that fluoride toothpaste consistently exhibited a notable decrease in symptoms associated with dentin hypersensitivity. Scholars conducted a study that demonstrated the efficacy of a toothpaste containing fluoride and potassium nitrate in lowering dentin hypersensitivity during a 12-week duration. Furthermore, dental associations and organizations consistently support the use of fluoride toothpaste as a dependable treatment choice for dentin hypersensitivity (DH).

Our investigation has established that abrasive toothpaste has a key role in causing dentin hypersensitivity. This discovery aligns with the prior study conducted by Petersson in 2013 [21], which also noted that the recession of the gumline, commonly caused by using toothpaste with abrasive properties, results in the exposure of the dentin around the tooth's root. The 2013 study conducted by Davari et al. [22] offers useful insights into the various impacts that toothpaste compositions might have on dentin hypersensitivity (DH). Their research emphasizes the significance of taking into account the precise constituents and strengths of desensitizing agents, together with other elements such as anti-plaque agents and abrasives, in toothpaste products. An important finding from their research is that toothpaste formulations might have contrasting impacts on dentin hypersensitivity, depending on their composition. The frequency of tooth brushing, particularly engaging in tooth brushing more than twice a day, is a crucial component of maintaining oral hygiene. Excessive tooth brushing, particularly when using abrasive toothpaste or employing forceful brushing techniques, has been proposed as a potential cause of dentin hypersensitivity or tooth sensitivity. Overzealous tooth brushing, especially when done more than twice a day, can potentially worsen or cause dentin hypersensitivity (DH) through various mechanisms such as enamel abrasion, gum recession, and tooth wear. Enamel abrasion can occur as a result of frequent and aggressive tooth brushing, particularly when using abrasive toothpaste. The enamel is the outer covering of the tooth that serves as a protective barrier. When the enamel is lost, it exposes the dentin underneath, which makes the tooth more vulnerable to external stimuli [23].

Besides that, gum recession can occur as a result of using forceful brushing techniques, causing the sensitive root surfaces of the teeth to become exposed. The roots' exposed dentin is extremely susceptible to dentin hypersensitivity (DH) [24]. Regular tooth brushing, when accompanied by the use of a highly abrasive toothpaste, can gradually lead to tooth wear. This can lead to the erosion of enamel and the uncovering of dentin, which can result in dentin hypersensitivity [25]. Although certain studies have investigated the correlation between regular tooth brushing and DH, further research is required to establish a definitive causal connection. Significantly, DH can also be impacted by a range of additional factors, such as food, oral hygiene practices, and preexisting dental diseases. The susceptibility of individuals to dentin hypersensitivity may differ, and it is not guaranteed that regular brushing will lead to the development of sensitivity in everyone. Oral health experts stress the significance of adhering to a regular oral hygiene regimen, which entails brushing teeth twice daily with a soft-bristle toothbrush and a nonabrasive toothpaste containing fluoride. To minimize the risk of enamel damage and gum recession, it is advisable to employ gentle brushing techniques and utilize a toothbrush with soft bristles.

The use of bootstrapping techniques in combination with ordinal regression is a significant methodological improvement in statistical analysis, especially in the field of medical research. Ordinal regression, a frequently used technique in medical research including ordinal data such as illness stages or pain levels, can be improved by incorporating bootstrapping. Bootstrapping is a resampling method that provides estimates of the sampling distribution [7,11].

The inclusion of bootstrapping in ordinal regression has greatly enhanced the accuracy of the model. Precision in statistical models is of utmost importance, particularly in the field of medical research, as erroneous forecasts might result in misguided treatment strategies. The bootstrapping technique enhances the accuracy of estimating model parameters by taking into account the underlying distribution of the data [26].

The main advantage of this approach is the decrease in standard errors. A reduced standard error in statistical modelling signifies more dependable and accurate forecasts [26]. The precision is of utmost importance in medical research, as the correctness of statistical models typically determines decision-making. A recent analysis has emphasized the significance of decreased standard errors in enhancing the reliability of medical research results. It has specifically highlighted bootstrapping as a technique for enhancing the accuracy of model estimations [27].

In addition, a study carried out by Alfons et al. [28] highlights the crucial significance of reduced standard errors in contemporary medical research. Their research showed that utilizing bootstrapping techniques resulted in decreased standard errors, which improved the accuracy of estimates and also increased the strength and replicability of findings. Ultimately, this enhanced the dependability of statistical analyses in the medical field. This emphasizes the increasing acknowledgment of bootstrapping as a helpful method for dealing with the difficulties related to standard mistakes in modern medical research.

This study assesses the efficacy of ordinal regression models in elucidating tooth sensitivity by employing diverse pseudo R-squared metrics. McFadden's pseudo R-squared, known for its tendency to provide conservative estimates in logistic regression models, has been calculated to be 0.548. This value indicates a substantial fraction of the variation in tooth sensitivity that can be explained by the model. The amount of explanatory capacity demonstrated aligns with the results of Rahim-Wöstefeld et al. [29], who reported comparable values in their investigations on tooth sensitivity. Significantly, when the number of replications escalates from 100 to 500, there is a marginal however constant enhancement in McFadden's pseudo R-squared and Nagelkerke's R-squared values, reaching a maximum of 0.608. This increase indicates an improvement in the accuracy of the model, which is consistent with the findings of Yuan and Gomer [30] regarding the resilience of ordinal regression when sample sizes are larger. Nevertheless, as the number of replications exceeds 500, a marginal decrease in the ability to explain is evident, prompting the need for additional inquiry. This occurrence, as proposed by Shmueli et al. [31], may be attributable to overfitting.

Similarly, the Cox and Snell (ML) pseudo R-squared begins at a higher initial value of 0.697, suggesting that a significant percentage of the variation in tooth sensitivity is accounted for. The stabilization of this value at 0.734 after 300 and 500 replications indicates a maximum level of improvement in explanatory ability with higher replications, which aligns with the theoretical limit provided by Cox and Snell. The observed pattern here supports the idea of diminishing returns when there are too many replications in model correctness. This topic was examined by Thompson and Wartenberg [32] in the context of ordinal regression models.

In addition, the Nagelkerke (Cragg and Uhler) R-squared, which offers a more reliable measure of the explained variance compared to a perfect model, indicates an initial value of 0.786. This demonstrates a significant level of explanatory capability in relation to tooth sensitivity, exceeding the threshold proposed by Nagelkerke for effective model performance. The model's explanatory power exhibited a steady improvement, reaching 0.828 over 500 replications. This upward trend aligns with the recent findings of Martin et al. [33] and Davis and Gift [34]. However, the decline to 0.804 after 1000 replications once again raises concerns regarding the possibility of overfitting or model saturation. This issue was emphasized by Park and Ho [35] in their research on regression models in health sciences.

In general, the ordinal regression model exhibits a high degree of explanatory capability for tooth sensitivity. Other models also exhibit gradual enhancements until a particular number of replications is reached. These findings enhance the current body of literature on the suitability and refinement of ordinal regression models in medical research, specifically in studies that investigate symptoms such as tooth sensitivity. Subsequent investigations could examine the consequences of these discoveries in medical environments, specifically in devising therapies for the control of dental sensitivity.

## Conclusions

This study highlights the significance of multiple variables such as age, toothpaste type, toothbrush type, and brushing frequency in understanding tooth sensitivity. Advanced statistical methods such as ordinal regression and bootstrap techniques enhance the model's accuracy. The findings suggest that specific oral hygiene practices can mitigate tooth sensitivity. This research provides valuable insights for dental health professionals in managing this condition. Practical guidelines are essential to reduce the risk of tooth sensitivity.

## References

[1] Addy M (2005). Tooth brushing, tooth wear and dentine hypersensitivity--are they associated?. Int Dent J.

[2] Irwin CR, McCusker P (1997). Prevalence of dentine hypersensitivity in a general dental population. J Ir Dent Assoc.

[3] Braennstroem Braennstroem, Astroem A (1964). A study on the mechanism of pain elicited from the dentin. J Dent Res.

[4] Orchardson R, Collins WJ (1987). Clinical features of hypersensitive teeth. Br Dent J.

[5] Porto IC, Andrade AK, Montes MA (2009). Diagnosis and treatment of dentinal hypersensitivity. J Oral Sci.

[6] Kumar S, Tadakamadla J, Johnson NW (2016). Effect of toothbrushing frequency on incidence and increment of dental caries: a systematic review and meta-analysis. J Dent Res.

[7] Agresti A (2010). Analysis of ordinal categorical data.

[8] Barnett V, Lewis T (1995). Outliers in statistical data. J Oper Res Soc.

[9] Adnan A, Sugiarto S (2017). The outlier detection for ordinal data using scalling technique of regression coefficients. J Phys Conf Ser.

[10] Agresti A (2013). Categorical data analysis. Alan Agresti. Stat Med.

[11] Efron B, Tibshirani RJ (1994). An Introduction to the bootstrap.

[12] Blaizot A, Offner D, Trohel G (2020). Prevalence of sensitive teeth and associated factors: a multicentre, cross-sectional questionnaire survey in France. BMC Oral Health.

[13] Ali AS, Varghese SS, Shenoy RP (2022). Association between cervical abrasion, oral hygiene practices and buccolingual dimension of tooth surfaces: a cross-sectional study. J Pharm Bioallied Sci.

[14] Yadav NS, Saxena V, Reddy R, Deshpande N, Deshpande A, Kovvuru SK (2012). Alliance of oral hygiene practices and abrasion among urban and rural residents of Central India. J Contemp Dent Pract.

[15] Sud N (2015). Prevalence of dental abrasion and its association with toothbrush frequency among patients attending O.P.D. in Government Dental College and Hospital - a cross sectional study. Indian J Dent Adv.

[16] Hegde M, Nireeksha Nireeksha (2015). Prevalence of tooth wear due to dietary factors in South Canara population. J Adv Med Med Res.

[17] Amin AH, Yaakob MH, Nasir WZ, Al-Kadhim AH (2018). Evaluation the correlation between age, gender, and the incidence of cervical lesions. Clin Res Dent.

[18] Haneet RK, Vandana LK (2016). Prevalence of dentinal hypersensitivity and study of associated factors: a cross-sectional study based on the general dental population of Davangere, Karnataka, India. Int Dent J.

[19] Bartlett DW, Fares J, Shirodaria S, Chiu K, Ahmad N, Sherriff M (2011). The association of tooth wear, diet and dietary habits in adults aged 18-30 years old. J Dent.

[20] Hu ML, Zheng G, Zhang YD, Yan X, Li XC, Lin H (2018). Effect of desensitizing toothpastes on dentine hypersensitivity: a systematic review and meta-analysis. J Dent.

[21] Petersson LG (2013). The role of fluoride in the preventive management of dentin hypersensitivity and root caries. Clin Oral Investig.

[22] Davari A, Ataei E, Assarzadeh H (2013). Dentin hypersensitivity: etiology, diagnosis and treatment; a literature review. J Dent (Shiraz).

[23] Ganss C, Schlueter N, Preiss S, Klimek J (2009). Tooth brushing habits in uninstructed adults--frequency, technique, duration and force. Clin Oral Investig.

[24] Gillam DG, Seo HS, Newman HN, Bulman JS (2001). Comparison of dentine hypersensitivity in selected occidental and oriental populations. J Oral Rehabil.

[25] West N, Addy M, Hughes J (1998). Dentine hypersensitivity: the effects of brushing desensitizing toothpastes, their solid and liquid phases, and detergents on dentine and acrylic: studies in vitro. J Oral Rehabil.

[26] (2008). Bootstrap methods: a guide for practitioners and researchers by Chernick, M. R.. Biometrics.

[27] Gupta S, Sedamkar RR (2020). Machine learning for healthcare: introduction. Machine learning with health care perspective: machine learning and healthcare.

[28] Alfons A, Ateş NY, Groenen PJ (2022). A robust bootstrap test for mediation analysis. Organ Res Methods.

[29] Rahim-Wöstefeld S, Kronsteiner D, ElSayed S, ElSayed N, Eickholz P, Pretzl B (2022). Development of a prognostic tool: based on risk factors for tooth loss after active periodontal therapy. Clin Oral Investig.

[30] Yuan KH, Gomer B (2021). An overview of applied robust methods. Br J Math Stat Psychol.

[31] Shmueli G, Bruce PC, Deokar KR, Patel NR (2023). Machine learning for business analytics: concepts, techniques, and applications with analytic solver data mining.

[32] Thompson WD, Wartenberg D (2007). Additive versus multiplicative models in ecologic regression. Stoch Environ Res Risk Assess.

[33] Martin GP, Mamas MA, Peek N, Buchan I, Sperrin M (2018). A multiple-model generalisation of updating clinical prediction models. Stat Med.

[34] Davis D, Gift T (2014). The positive effects of the Schengen agreement on European trade. World Econ.

[35] Park Y, Ho JC (2021). Tackling overfitting in boosting for noisy healthcare data. IEEE Trans Knowl Data Eng.

